# To Increase Our Knowledge on Sleep, Sleep Disorders, and Chronobiology in the Neuroscience Field during the Next Decade

**DOI:** 10.3389/fneur.2017.00016

**Published:** 2017-02-09

**Authors:** Yves Dauvilliers

**Affiliations:** ^1^Sleep-Wake Disorders Center, Department of Neurology, Hôpital Gui-de-Chauliac, INSERM U1061, CHU Montpellier, National Reference Network for Narcolepsy, Idiopathic Hypersomnia and Kleine Levin Syndrome, Montpellier, France

**Keywords:** sleep, sleep disorders, neurology, chronobiology, challenges, narcolepsy, sleep–wake regulation, circadian systems

The Grand Challenge for Sleep and Chronobiology in the Neurology/Neuroscience domains for the twenty-first century is to improve our understanding of the complex relationships between sleep, wake, and chronobiology in healthy subjects and in patients with neurological disorders.

Sleep disorders are very common and often disabling conditions that can be isolated or associated with neurological disorders. In addition to neurologists and neurophysiologists, pulmonologists, psychiatrists, cardiologists, pediatricians, ENT doctors, psychologists, geneticists, and basic scientists also are involved in sleep disorder diagnosis and research. This corresponds to a heterogeneous group of physicians/researchers with different interests and expertise that include basic and clinically applied research. Our diversity of interests is both our strength and a challenge.

Sleep is a universal physiological need that plays a key role in the prevention of health problems. In modern life, sleep disruption through behavioral deprivation is a major issue. Indeed, one-third of the population is affected by sleep disorders, as defined by the revised International Classification of Sleep Disorders (1), namely insomnia, central hypersomnolence disorders (such as narcolepsy), sleep-related movements disorders (such as restless legs syndrome), sleep-related breathing disorders, parasomnias (such as sleepwalking and REM-sleep behavior disorder), and circadian rhythm sleep–wake disorders. Epidemiological studies have demonstrated the large prevalence of many sleep disorders in the general population, especially in patients with neurological and psychiatric diseases, and their association with a decline of cognitive performance, productivity, and quality of life, with a major social, medical, and economic impact. People with sleep disorders are often at risk of accidents, psychiatric, neurological and cardiovascular diseases, obesity, and metabolic disorders, for example, sleep-disordered breathing, excessive daytime sleepiness, short and long sleeping time are risk factors for cardiovascular diseases; insomnia for depression; REM-sleep behavior disorder for Parkinsonism, etc. In addition, sleep–wake alterations (e.g., reduced slow wave sleep and circadian disturbances) may influence the course and outcome of neurodegenerative diseases, such as Alzheimer’s disease (2).

Narcolepsy with cataplexy remains the classic example of neurological sleep disorder and one of the most studied sleep disorders at the molecular level. Narcolepsy is a disabling disorder characterized by excessive daytime sleepiness and abnormal rapid-eye-movement sleep manifestations, including cataplexy (sudden loss of muscle tone triggered by strong emotions), sleep paralysis, hypnagogic hallucinations, and sleep onset REM periods (3). Narcolepsy with cataplexy has been renamed narcolepsy type 1 due to the deficiency in hypocretin signaling caused by the selective loss of hypothalamic hypocretin neurons. Conversely, no other specific reliable biomarker has been described so far for most of the other sleep disorders.

Sleep and wakefulness are complex behaviors regulated at many different levels that are likely to be under strong environmental, immune, and genetic control (4). In the past decades, clinical and translational studies on sleep and circadian systems provided exciting insights for the entire field of neurobiology, sleep–wake processing, regulation, and function. The combination of increased technical complexity and growing need of interdisciplinary research poses new challenges to our community with contributions from many scientific fields, such as neuroscience, physiology, biology, genetic, epidemiology, psychology, and medicine. Thus, advances in translational research provide key opportunities to explore sleep physiological and pathological effects in different neurological diseases. However, the great progress in our understanding of the physiological sleep–wake and circadian processes has only slightly modified our diagnostic procedures and management of sleep disorders (5).

We need to improve the diagnosis of sleep disorders for their early detection and better management and also for the identification of sleep–wake alterations as risk factors of bad outcome of neurological conditions. The treatment of sleep disorders may have positive consequences on disease progression, for instance using continuous positive air pressure in patients with severe obstructive sleep disorders breathing. Further interventional studies are needed to determine whether treatment of sleep disorders might improve neurological disorders, prevent their development and progression, and whether their optimal management might improve sleep in these populations.

The Grand Challenges for Sleep and Chronobiology in Neurology/Neuroscience are to improve our understanding of sleep–wake and circadian regulation and function; to disseminate the knowledge on sleep and sleep disorders to pre- and post-graduate physicians; and to educate neurologists and psychiatrists who, in the past, often neglected sleep as well as neuroscience researchers. Finally, we need to discover better treatment and to define up-to-date guidelines for the management of patients with primary or comorbid sleep disorders.

We hope that the *Frontiers in Neurology* “Sleep and Chronobiology” section will become one of the first choices for sleep-related publications by clinicians and researchers interested in this field. Our goals are to publish open-access high-quality research with rapid publication to advance our understanding of this large and complex discipline. We accept various manuscript types and strongly encourage submission of original articles.

## Author Contributions

The author confirms being the sole contributor of this work and approved it for publication.

## Conflict of Interest Statement

The author declares that the research was conducted in the absence of any commercial or financial relationships that could be construed as a potential conflict of interest.
